# Creating realistic nerve agent victim profiles for computer simulation of medical CBRN disaster response

**DOI:** 10.3389/fpubh.2023.1167706

**Published:** 2023-06-29

**Authors:** Ruben De Rouck, Mehdi Benhassine, Michel Debacker, Christian Dugauquier, Erwin Dhondt, Filip Van Utterbeeck, Ives Hubloue

**Affiliations:** ^1^Research Group on Emergency and Disaster Medicine, Vrije Universiteit Brussel, Brussels, Belgium; ^2^Department of Mathematics, Royal Military Academy, Brussels, Belgium; ^3^Twenty-third Medical Battalion, Belgian Defence, Tournai, Belgium; ^4^Belgian Delegate in The NATO Biological Medical Panel, Brussels, Belgium; ^5^DO Consultancy, Brussels, Belgium

**Keywords:** victim model, injury profile, organophosphates, nerve agent, sarin, computer simulation, disaster preparedness

## Abstract

In the last decades, Chemical, Biological, Radiological and Nuclear (CBRN) threats have become serious risks prompting countries to prioritize preparedness for such incidents. As CBRN scenarios are very difficult and expensive to recreate in real life, computer simulation is particularly suited for assessing the effectiveness of contingency plans and identifying areas of improvement. These computer simulation exercises require realistic and dynamic victim profiles, which are unavailable in a civilian context. In this paper we present a set of civilian nerve agent injury profiles consisting of clinical parameters and their evolution, as well as the methodology used to create them. These injury profiles are based on military injury profiles and adapted to the civilian population, using sarin for the purpose of illustration. They include commonly measured parameters in the prehospital setting. We demonstrate that information found in military sources can easily be adjusted for a civilian population using a few simple assumptions and validated methods. This methodology can easily be expanded to other chemical warfare agents as well as different ways of exposure. The resulting injury profiles are generic so they can also be used in tabletop and live simulation exercises. Modeling and simulation, if used correctly and in conjunction with empirical data gathered from lessons learned, can assist in providing the evidence practices for effective and efficient response decisions and interventions, considering the contextual factors of the affected area and the specific disaster scenario.

## Introduction

Health is recognized by the Sendai Framework for Disaster Risk Reduction (SFDRR) 2015–2030 as an important component of disaster risk reduction. It specifically considers the enhancement of disaster preparedness of the health systems and the training capacities in the field of disaster medicine as one of the priorities of the disaster risk reduction strategies. Moreover, the SFDRR 2015–2030 emphasizes the development of the evidence base and the increase of the scientific and technical impact on disaster risk reduction, including modeling studies to assess exposure to all hazards (1). Managing mass casualties in a disaster situation or humanitarian crisis can no longer rely on goodwill and good intentions. An evidence-based practice is needed to achieve the objectives of the health disaster management due to the immediate impact on the community and especially on the healthcare system, the number and variety of injured or ill victims, an initial phase of disorder, the temporary lack of resources and limited output of medical teams directly after the disaster, the necessity to operate in multidisciplinary and complementary teams, and the multiplicity of tasks (2). Just as evidence-based decision making has gained momentum in medical research, simulation has emerged over the last decades as a useful tool in the study of disaster preparedness. Traditional analytic methods cannot fully capture the flow of disaster victims through a complex health disaster response system (3).

In contrast to discussion-based, tabletop or live exercises, computer modeling, and simulation can enhance disaster preparedness by studying all operational assumptions and testing contingency plans in a virtual but controlled experimental environment. Simulation allows the integration of stochastic and dynamic aspects inherent to the health disaster response and to study possible relationships among any or all variables included in the scenario (4, 5). Computer-generated victims can be created with vital parameters that are realistic for their injuries and with a randomly allocated severity of injuries based on probabilities for the different triage classes (6, 7). The health status of the victims can be adapted to time and treatment or lack of treatment (8).

In the last decades, Chemical, Biological, Radiological and Nuclear (CBRN) threats have become serious risks prompting countries to prioritize preparedness for such incidents (9). Numerous studies have highlighted worldwide serious gaps in the preparedness of healthcare providers to cope with CBRN incidents (9–11). Managing CBRN emergencies is substantially different to routine interventions of healthcare workers. Moreover, performance can seriously be reduced by stress and urgency generated by CBRN incidents (7). The expertise of CBRN responders has therefore an important impact on the mortality and morbidity rates of victims (12). However, rapid interventions can reduce the harmful effects of CBRN emergencies, requiring a training in the health aspects of CBRN agents (9).

As CBRN scenarios are very difficult and expensive to recreate in real life, computer simulation is particularly suited for assessing the effectiveness of contingency plans and identifying gaps and areas of improvement (5, 13). Simulation techniques allow healthcare providers to investigate the impact of different options and thus provide evidence that decision makers need in order to establish robust response strategies in the CBRN area. Sensitivity analysis may determine the extent of resources to meet the needs for managing CBRN emergencies (8).

## Methods

### Victim profiles

We set out to create a set of victim profiles based on the nerve agent sarin (GB) for use in computer simulation. We specifically chose the nerve agent sarin due to historical significance as well as the wide body of available research in the literature (14–17). A literature study failed to identify victim profiles suitable for use in a civilian population. However, we identified a set of similar victim profiles in the – now superseded – North Atlantic Treaty Organization (NATO) Standardization Agreement (STANAG) 2,553 covering the Allied Medical Publication 8(C), the NATO Planning Guide for the Estimation of CBRN Casualties (AMedP-8(C)). This AMedP-8(C) describes a set of 6 victim profiles of increasing severity in symptomatology, each linked to a specific inhalation exposure interval. These military injury profiles specifically exclude the effects of treatment or medical countermeasures such as pre-exposure prophylaxis. They were created by NATO Subject Matter Experts (SMEs) incorporating available evidence from human and animal exposures. While the profiles were originally with the intended purpose of casualty estimation, our goal is to develop victim profiles that can also be used to estimate victim severity distributions, resource requirements and to train healthcare workers.

Every AMedP-8(C) victim profile consists of 6 separate symptom severity evolutions. Every category is assigned a numerical value representing severity of compromise, ranging from 0 representing no symptoms up to 4 representing severe (life-threatening) compromise. Every category has a detailed description assigned to each severity value used. The categories described in the AMedP-8(C) publication are ocular (0–3), upper-gastrointestinal (0–2), lower-gastrointestinal (0–3), respiratory (0–4), muscular (0–4) and neurological (0–4) symptoms. Clinical evolution over time is described by the timing of the improvement of these categories (18).

Injury profile 1 (IP-1) represents a very mildly intoxicated victim, only showing ocular symptoms, and spontaneously recovering after 6 h (or more quickly with adequate treatment). Injury profile 2 also represents a mildly intoxicated but a more severely intoxicated victim than IP-1. Injury Profile 2 (IP-2) victim exhibits respiratory symptoms (dyspnea and wheezing) for 2.5 h and spontaneously recovering ocular symptoms persisting over a week. Injury profile 3 (IP-3) represents a moderately intoxicated victim with a combination of mild gastro-intestinal and mild respiratory symptoms, which improve progressively after 1 to 2 days. Injury profile 4 (IP-4) represents a moderately intoxicated victim with severe respiratory distress and bronchorrhea. Victims belonging to IP-4 are expected to manifest severe respiratory insufficiency within the first 5 min after exposure. This is due to an irregular respiratory rate in combination with severe muscular fatigue. Both improve progressively after 6 to 16 h. Injury profile 5 (IP-5) represents a severely intoxicated victim and sustains a very severe intoxication with brief self-limiting seizures (and secondary respiratory arrest). Their condition is expected to spontaneously improve within 10–15 min after exposure. The IP-5 clinical condition improves progressively over the next 6 to 16 h, taking well over a week to return to baseline. Injury profile 6 (IP-6) represents the most severely intoxicated victims. They quickly show most severe symptoms because of respiratory insufficiency of combined neurological and origin. In AMedP-7.5, victims conforming to this profile are assumed to perish after 15 min when left untreated. However, when receiving adequate antidote treatment and respiratory support, they are expected to survive. Table 1 contains a brief description of the injury profiles adapted from AMedP-8(C).

**Table 1:** Brief overview of symptoms and timing for each of the injury profiles.

Injury profile	Description
IP-1	Brief episode of ocular symptoms (pain and miosis) only. Spontaneous recovery after 6 h. Exposure range: 0,2 to 1 mg min m^−3^.
IP-2	Mild ocular and mild respiratory symptoms (wheeze and dyspnea). Respiratory symptoms improve after 1.5 h, and ocular symptoms improve after + − 16 h but linger for weeks. Exposure range: 1 to 6,5 mg min m^−3^.
IP-3	Moderate intoxication with mild GI and respiratory symptoms. These symptoms last about a week. Ocular symptoms persist longer. Exposure range: 6,5 to 12 mg min m^−3^.
IP-4	Moderate intoxication with severe bronchorrhea, respiratory distress and mild neurological impairment (agitation, anxiety, twitching, and convulsions). Improvement after 60–90 min, but mild ocular, respiratory and GI symptoms persist for days to weeks. Exposure range: 12–25 mg min m^−3^.
IP-5	Severe intoxication with respiratory insufficiency (central, muscular, and due to secretions), seizures and severe ocular and GI symptoms. Brief seizures/coma (+ − 15 min) but severe respiratory, muscular, and neurological symptoms persist for 1–2 h, slowly improving over days to weeks. Exposure range: 25–30 mg min m^−3^.
IP-6	The most severe intoxication, where all symptoms are of the most severe category. Death is expected after 15 min if untreated, due to a combination of flaccid paralysis, respiratory insufficiency, and status epilepticus/coma. Exposure range: over 30 mg min m^−3^.

### Assignment of clinical parameters

Clinical decision making and therefore resource estimation is based on the patient’s general condition and physiological parameters. Concise data on human clinical parameters of nerve agent exposures is very rarely reported in the detail required. Usually, these parameters are reported in general terms without clarification, such as bradycardia, tachycardia, confusion and decreased consciousness. The clinical parameter assignment algorithm is based on subject matter experts and victim profiles developed use in military and civilian training exercises (19, 20). We attempted to validate the model by linking the reported parameters to injury profiles and time of exposure, but it should be noted that these simulation victims are also mainly expert opinion based and are inspired by organophosphate poisoning and do not quite correspond to victim descriptions from the Tokyo 1995 subway attack. For instance: bradycardia and hypotension are only described in a preterminal phase, and respiratory hypersecretions are less common (21–23).

Using this information, a set of conversion rules are devised to convert the severity categories to clinical parameters. To create clinically relevant profiles, our choice of clinical parameters is limited to those measurable and treatable in a prehospital setting. The selected parameters are airway status, respiratory rate, respiration depth, respiratory rate, oxygen saturation, heart rate, blood pressure, Glasgow Coma Scale, and pupillary status. Unfortunately, these parameters are at most slightly hinted at in the description of the Injury Profiles in AMedP-8(C). We therefore created a set of simple algorithms to uniformly convert the severity categories and their interactions to clinical parameter ranges. The specific algorithms used are described in appendix 1.

Airway status is based on the presence of severe respiratory and motor incapacitation, and ranged from normal, snoring to obstruction. Obstruction of the airway is assumed to be present when either the neurological or motor incapacitation reaches the worst severity.

Mild respiratory involvement is described as having only mild shortness of breath, chest tightness, coughing and a runny nose. The patient becomes dyspneic when the respiratory incapacitation becomes moderate or severe, due to the increase in secretions. Very severe respiratory incapacitation leads to shallow respirations due to hypoxia secondary to secretions in combination with flaccid paralysis. We estimate that the respiratory rate will first rise to 20–30 respirations per minute when the mild respiratory impairment is modeled, increasing to 30+ with moderately affected respiration. Severe respiratory incapacitation will lead first to an increased respiratory rate and then to a decrease due to respiratory fatigue and intermittent apnea. Finally, the very severe respiratory impairment results in flaccid paralysis and secondary apnea.

Oxygen saturation modeling is based on the categories defined by Raux et al.: no respiratory incapacitation corresponds to an oxygen saturation of 95%–100%. We assume a mild impairment results in an oxygen saturation of 90%–95%. Moderate impairment is assumed to result in a saturation of 85%–90%. Severe respiratory insufficiency corresponds to a saturation of 80%–85% and with very severe respiratory impairment corresponds to the tipping point where the patient can no longer sustain their own oxygenation, resulting in desaturation below 80% (24).

There is no mention of heart rate or blood pressure in the AMedP-8(C) injury profiles. The goal of these profiles is to provide realistic profiles for the general population, which has a large degree of uncertainty. We therefore chose not to report exact numbers and opted for relative values instead. Sarin and other G-agents have been described as having very strong nicotinergic effects on the cardiovascular system, leading to hypertension and tachycardia even in severe intoxications (25). Bradycardia and hypotension are therefore only expected as very late signs of the most severe intoxication due to a terminal hypoxic state, so they are assumed only to be present when respiratory and neurological impairment is so overwhelming that it causes death. In practice, this means that it is only assumed to be present in IP-6.

There is not enough information available in the description of the neurological category of the Injury Profiles to correlate the full Glasgow Coma Scale (GCS) based on the description alone. Toxicological Physiologically-based Pharmacokinetic/Pharmacodynamic (PBPK/PD) modeling has proven useful in modeling symptoms and NA countermeasures (26). Rodriguez and McClellan developed such a model for inhalational sarin battlefield exposures, using data from human and animal exposures found in the literature. It incorporates probability of mortality and the effect of atropine and/or oxime administration and bioscavengers on mortality, based on a modeling of the whole-body stimulated acetylcholinesterase receptor fraction by the sarin (27). This model has been extensively validated by comparison to existing models and physiological constants and its predictions of untreated progressions correspond very well to the neurological categories predicted by the AMedP-8(C) after a 1-min inhalational exposure.

While the level of detail in the injury profile is scarce, certain relationships can be inferred. For instance, a neurological category of 4 corresponds to E1V1M1. Severe neurological compromise corresponds to V4 or lower. Mild and moderate neurological compromise. For the motor component of the GCS there are no relationships described in the injury profile description, but we can assume that a flaccid paralysis will lead to a value of 1. For the eye-opening component of the GCS, we assume that severe ocular involvement will prevent eye opening due to antalgic blepharo- and ciliary spasms, corresponding to an E value of 2. Miosis is described starting at mild ocular involvement. GCS Values that cannot be reliably linked to the level of neurological compromise are represented by an interval.

### Extrapolation of exposure levels to the general population

To adapt the exposure intervals of AmedP-8(C) to a probit model, we referenced NATO AMedP-7.5, Edition A, Version 1, NATO Planning Guide for the Estimation of CBRN Casualties (October 2017) – which supersedes the aforementioned AmedP-8(C). It describes a methodology to estimate the proportion of a population affected by chemical and biological threats, for the purposes of casualty estimation. In this publication the authors present a probit model, as well as a methodology to apply it to the AMedP-8(C) injury profiles (28). Equation one is used to estimate the probability of an individual conforming to an injury profile assuming a normal healthy 70 kg victim with a respiratory rate of 15 liters per minute.


(1) $$P\left(IP\right)=\Phi \left(\;PS\cdot \log_{10}\left(\;\frac{Exposure\;}{ECt50}\right)\right)$$

In this equation, the ECt_50_ represents the exposed concentration time (measured in mg.min.m^−3^) for which half the population will be affected in the way described by the injury profile (or worse). We assume this to be the mathematical average of the exposure range interval. The lack of upper bound on the dosage range for IP-6 creates a problem for this assumption. In the original AMedP-8(C) publication it is reported that the LD50 of sarin is located within the IP-6 range, implying that not all victims exposed to this range will be lethally injured. Since the development of AMedP-7.5 the LD50 value was decreased to 33 mg.min.m^−3^. The probit model is centered around the ECT_50_ value, which together with the assumption that IP-6 is a lethal profile, means the LD_50_ value can be used as the ECt_50_.

These ECt_50_values assume a body weight of 70 kg and a minute ventilation of 15 liters per minute. The probit slope (PS) is a parameter that represents the genetic and physiological variability in the response to the toxic agent. The lower the PS, the more varied the effects of an exposure. The total exposure is calculated as an aggregate concentration-time product (ECt) and is also expressed in mg.min.m^−3^.

Equation 1 returns a probability of an injury level being at least as severe as the IP. One can calculate the IP specific probability by subtracting the probability that a victim conforms to a worse IP. For example: if a victim is calculated to have a probability of 70% of presenting as IP-5 and a 40% probability of presenting as IP-6, then the final probability of the victim presenting as IP-5 is 30% and as IP-6 is 40%.

**Figure 1:**
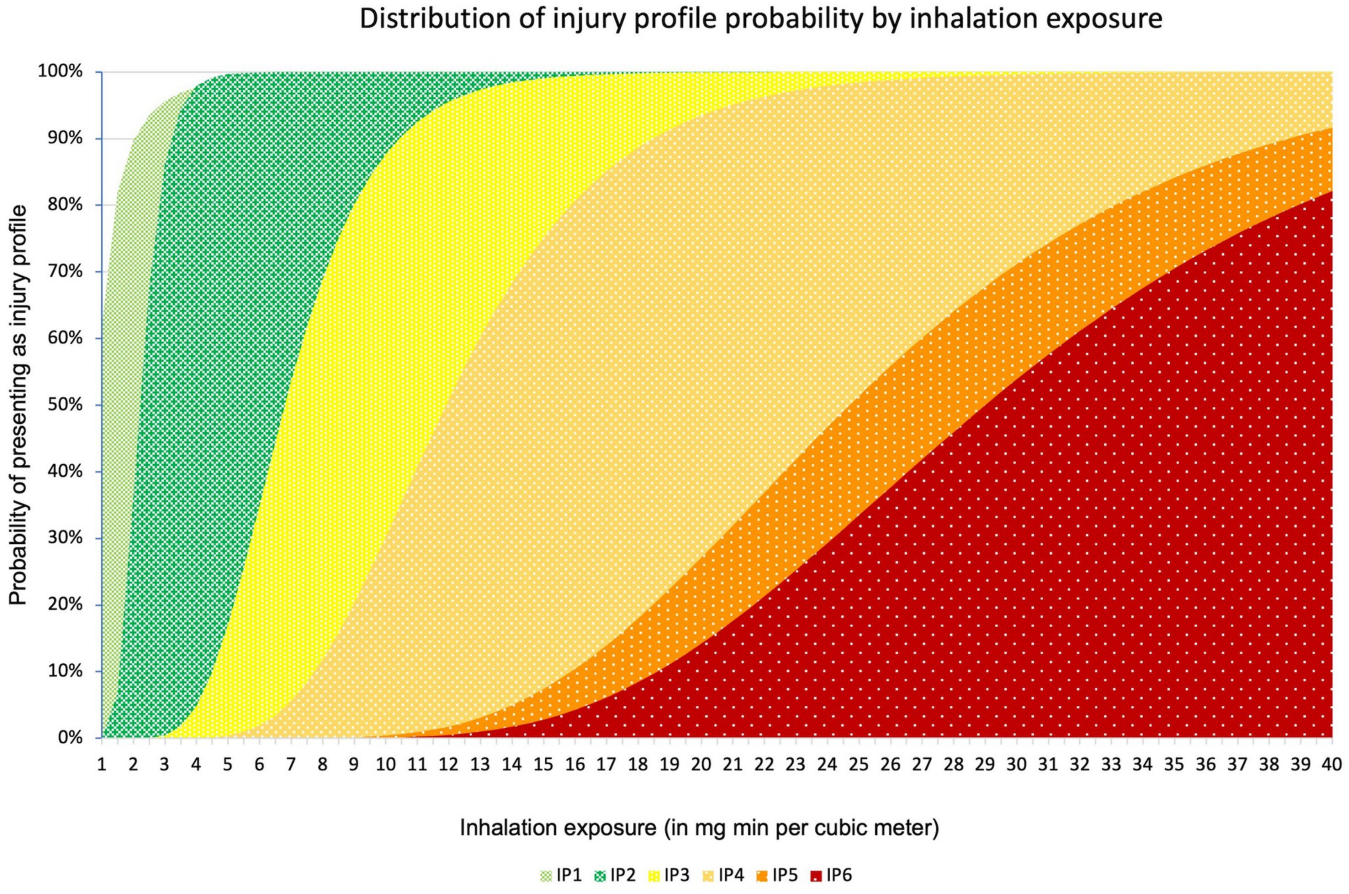
Distribution of injury profile probability by concentration time. On the X-axis the exposure is expressed in mg min m^−3^. On the Y axis the cumulative probability can be found.

To convert the military exposure ranges from the injury profiles to a general population, we used the extrapolation method described by Crosier and Somerville, assuming a normal population distribution for both military and civilian population (29). This method assumes that the military population consists of the healthiest 25% of the general population. They estimated an uncertainty factor based on either the top 25% of the bell curve (tail model), or a bell curve within a bell curve (bell model). The uncertainty factor for conversion of mean toxicity values between military and general population is estimated between 1.4 and 1.57 for a bell model and tail model, respectively. Similarly, the probit slope of 12 is widened to 5.9 and 7.2 for a bell model and tail model, respectively, for non-mild inhalational G-agent exposure. In the case of mild inhalation exposure, the resultant probit slope is 2.22 and 2.71, respectively, for a bell and tail model, resulting in an average probit slope of 2.5. Because neither bell nor tail model offers a theoretical benefit and the results for both models are comparable, we chose to use the average of both models for our final exposure range calculations. The resultant ECt_50_ exposure intervals and PS parameters are presented in Table 2. A graphic depiction of the resultant distributions can be found in Figure 1.

**Table 2:** Overview of injury profile mathematical model parameters.

Parameter	IP-1	IP-2	IP-3	IP-4	IP-5	IP-6
Lower Ct_mil_	0.2	1	6.5	12	25	30
Upper Ct_mil_	1	6.5	12	25	30	>30
PS_mil_	4.5	4.5	12	12	12	12
ECt_50,mil_	**0.**.**6**	**3.75**	**9.25**	**18.5**	**27.5**	**33**
Lower Ct_gen_	0.14	0.68	4.4	8	17	20
Upper Ct_gen_	0.68	4.4	8.11	17	20	N/A
PS_gen_	2.5	6.5	6.5	6.5	6.5	6.5
ECt_50,gen_	**0.35**	**1.7**	**6.3**	**11.4**	**24.2**	**28.5**

In this table the resulting values for the extrapolation of the 6 injury profiles are given. The first two rows contain the exposure interval bounds of the military population expressed in mg.min.m-3. Rows 5 and 6 contain the equivalents of the general population. Rows 3 and 4 contain the PS and ECt50 parameters for the military population, while rows 7 and 8 contain the PS and ECt50 parameters for the general population.The bold values in table represent the calculated ECT50s by the methodology for the military and for the general population respectively.

## Results

Table 3 displays the resulting clinical parameters and progression over time associated with every injury profile, using the algorithms described above.

**Table 3:** Overview of the individual victim profiles and their physiological parameter progression over time.

Time (minutes)	Airway	RR	SaO2	HR	SBP	GCS	Pupil size
*Injury profile 1*							
1	Normal	10–20	95–100	60–100	90–120	15	Normal
5	Normal	21–29	90–100	60–100	90–120	15	Miosis
360	Normal	10–20	95–100	60–100	90–120	15	Normal
*Injury profile 2*							
1	Normal	10–20	95–100	60–100	90–120	15	Normal
3	Normal	21–29	90–100	60–100	90–120	13–15	Miosis
152	Normal	10–20	90–100	60–100	90–120	15	Miosis
1002	Normal	10–20	95–100	60–100	90–120	15	Miosis
*Injury profile 3*							
1	Normal	10–20	95–100	60–100	90–120	15	Normal
3	Normal	21–29	90–100	60–100	90–120	13–15	Miosis
5	Normal	21–29	90–100	>120	>120	13–15	Miosis
14	Normal	21–29	90–100	>120	>120	13–15	Miosis
1944	Normal	10–20	95–100	>120	>120	15	Miosis
*Injury profile 4*							
1	Normal	10–20	95–100	60–100	90–120	15	Normal
3	Normal	21–29	90–100	60–100	90–120	13–15	Miosis
5	Normal	21–29	90–100	>120	>120	13–15	Miosis
7	Snoring	5–15	90–85	>120	>120	6–14	Miosis
21	Normal	>30	90–100	>120	>120	6–14	Miosis
66	Normal	>30	90–100	>120	>120	13–15	Miosis
106	Normal	>30	95–100	>120	>120	13–15	Miosis
1006	Normal	10–20	95–100	>120	>120	13–15	Miosis
8646	Normal	10–20	95–100	>120	>120	15	Miosis
*Injury profile 5*							
1	Normal	10–20	95–100	60–100	90–120	15	Normal
3	Normal	21–29	95–100	60–100	90–120	13–15	Miosis
5	Normal	21–29	95–100	>120	>120	13–15	Miosis
7	Snoring	5–15	90–85	>120	>120	6–14	Miosis
14	Snoring	5–15	90–85	>120	>120	3	Miosis
23	Snoring	5–15	90–85	>120	>120	6–14	Miosis
68	Normal	>30	90–100	>120	>120	6–14	Miosis
158	Normal	>30	90–100	>120	>120	13–15	Miosis
248	Normal	21–29	95–100	>120	>120	13–15	Miosis
1008	Normal	21–29	95–100	>120	>120	13–15	Miosis
1948	Normal	10–20	95–100	>120	>120	13–15	Miosis
8648	Normal	10–20	95–100	>120	>120	13–15	Miosis
20168	Normal	-20	95–100	60–100	>120	13–15	Miosis
*Injury profile 6*							
1	Normal	10–20	95–100	60–100	90–120	15	Normal
3	Normal	21–29	95–100	60–100	90–120	13–15	Miosis
5	Normal	21–29	95–100	>120	>120	13–15	Miosis
7	Snoring	5–15	90–85	>120	>120	6–14	Miosis
11	Obstructed	<5	<80	<60	<90	3	Miosis
26	**Victim is presumed dead if untreated**						

RR, respiratory rate in respirations per minute; SaO2, oxygen saturation in percent; SBP, systolic blood pressure in mmHg; GCS, glasgow coma scale.

## Discussion

This methodology uses the most recent evidence available from respected and publicly available sources to create a set of injury profiles for simulation. It accounts for the genetic and physiological variation in response to OP exposure through stochastic variation using values compatible with the general population derived from solid assumptions. The described approach is applied to inhalational sarin exposure but can be extrapolated to other chemical warfare agents (CWAs) and other exposure routes by modifying the agent specific parameters and the timelines accordingly. We chose to neglect cutaneous exposure in the modeling of these specific injury profiles because they are several orders of magnitude greater than the inhalational exposure required to produce a similar effect. One of the prerequisites for assigning the victim’s injury profile is an estimation of its exposed concentration time integral. These estimation methods are available in the AMedP-7.5 (30).

The values calculated above using the presented method conform to the data reported in the literature. For example, Bide et al. used a bivariate surface model to estimate human toxicity from animal exposures, for which they report a lethal concentration for 50% of the population of 36 for an exposure of 2 min, as well as a slightly wider probit slope of 4.5 (31). However, they also state that their methodology might not be optimally suited for probit slope calculations due to selection bias and factors inherent to the animals used.

We intend to use these injury profiles as part of a discrete event simulator called SIMEDIS, used to analyze the disaster medical response chain (8, 32). These injury profiles are included in the building of a continuous victim model, where the evolution of a victim’s health state is modeled over time as a categorical sum of the heart rate, GCS, respiratory rate, systolic blood pressure and oxygen saturation (33).

### Modeling assumptions

The values presented in Table 2 are valid for inhalation exposure only. A similar methodology can be applied to the PS and ECt50 values of cutaneous exposure, as well as different times due to the delayed absorption.

Despite using available evidence, all injury profiles were initially created by SMEs and therefore are susceptible to bias. These injury profiles do not include the delayed or chronic effects such as OP induced peripheral neuropathy or OP intermediate syndrome. The victim profiles do not include treatment effects nor the timing of administration of treatment regarding the aging effect. There is no modeling of the effect of decontamination. If one were to model the effects of decontamination, one would have to include the method of exposure, absorption mechanics of the agent and the effectiveness of decontamination to remove the agent. To our knowledge there are no published reports on either of these properties for sarin. The exposure intervals are valid for very short exposures, assume no respiratory or pharmacological protection and are valid for individuals weighing 70 kg. To our knowledge there is no data available on differences between sexes. The values also assume the applicability of Haber’s law. Haber’s law is a toxicological concept that states that the concentration-time product required for a set of symptoms is a constant. This means that a long exposure to low concentrations and a short exposure to high concentrations should theoretically result in similar symptoms (34). Because Haber’s law has been repeatedly demonstrated not to apply to CWAs, a toxic load exponent model is proposed for longer exposures, to compensate for metabolization of organophosphates by increasing the dose required to attain the intoxication level described by the injury profiles as a function of time. Bide et al. calculated this toxic load exponent to be 1.4, based on extrapolation from multiple animal exposure experiments, for exposures up to 30 min (31).

The AMedP-8(C) injury profiles start after victims have received their full exposure. These injury profiles assume that the exposure happens over a relatively short (<1 min) timeframe and the exposure ends before the victim clinical evolution starts. Discussions with SMEs and data from a Rodriguez and McClellan’s PBPK/PD model suggest that there will be a time delay of 1–2 min per injury profile before the sarin has reached adequate distribution and receptor binding for victims to exhibit their maximum symptoms. Methods of dispersion and release are outside the scope of this publication. However, slower exposures will result in an even more pronounced progressive degradation of the clinical condition.

We would also like to point out that the estimation of exposure is considered outside the scope of this work due to additional complexities. There are several methods available, from simple gaussian puff models to the computationally intensive but current gold standard of computational fluid dynamics (35–37). We refer the reader to the AMedP-7.5 publication for more details on specific exposure settings, such as the effects of physical exercise and respiratory protection (30).

### Special considerations

Treatment of these injuries is outside the scope of this publication, but discussed here Nerve agent treatment classically consists of the antimuscarinic agent atropine to reduce secretions and an oxime to reactivate the acetylcholinesterase (38). Incidental seizures are treated with benzodiazepines which are GABA-A receptor agonists and NMDA receptor antagonists such as ketamine when they become ineffective due to GABA-A receptor downregulation or when sedation is required (39–41). In the case of sarin, the oxime of choice is pralidoxime (42, 43). Oximes are classically thought to be best administered within 1 h of exposure, however continued administration starting after 1 h has shown to still have significant beneficial effects (44). The modeling of the effect of these agents is beyond the scope of this work but reports from the 1995 Tokyo attack show a variable response to atropinization: both an increase and a paradoxical decrease of heart rate and systolic blood pressure have been reported. Seizures were successfully treated using diazepam titration. Oxime administration and an increase of plasma cholinesterase levels were associated with improvements in respiratory conditions and normalization of miosis (21–23). Reports and theoretical models show that exposures of up to threefold the LD50 values are considered survivable with rapid and adequate treatment and frequent re-administration of antidotes (27, 45). Nerve agent exposure is also described to leave lasting effects such as OP-ester induced delayed neurotoxicity. These effects should be considered in victims of nerve agent exposure but are not modeled here as they do not alter the modeled parameters in a significant fashion. It is however assumed that early oxime administration lowers the probability of the development of long-term neurological side effects (41).

Decontamination and secondary contamination are outside the scope of this work due to the lack of available data. Retrospective reports of the Japanese sarin attacks report moderate secondary contamination of health care workers in closed rooms (46). When designing a simulation exercise, close attention should be paid to the scenario to assess the effects of decontamination on delayed absorption. The exposure route as well as dispersion method can lead to significant differences in the symptomatology and onset thereof, as well as secondary contamination of health care providers (47).

Possible applications of these injury profiles are serving as a basis to create scenarios of a sarin (or other nerve agent) mass casualty incident, as well as for creating realistic victims or determining the injury severity distributions of victims for an assumed exposure. This methodology has already been successfully implemented in a computer simulation model of an urban subway station chemical attack, as well as a live decontamination and victim reception exercise in a tertiary care hospital (48, 49).

## Conclusion

Modeling and simulation, if used correctly, in conjunction with empirical data gathered from lessons learned, can assist in providing the evidence practices for effective and efficient response decisions and interventions, considering the contextual factors of the affected area and the specific disaster scenario. In this paper a methodology is presented to create a set of realistic injury profiles and severity distributions for organophosphate chemical warfare agent victims for known exposures. Not included in this work are the effects of decontamination and secondary contamination, the progression of treated victims and long-term effects.

## Data availability statement

The original contributions presented in the study are included in the article/Supplementary material, further inquiries can be directed to the corresponding author.

## Author contributions

RD is the principal author of this work. MB, FV, MD, and IH made contributions to the article and supervised the work. ED and CD acted as military SMEs. All authors contributed to the article and approved the submitted version.

## Funding

MB is supported by the Department of Scientific and Technological Research of Defence of the Royal Higher Institute for Defence (Belgium; grant no. HFM21-12).

## Conflict of interest

ED is employed by and the owner of DO Consultancy BV.

The remaining authors declare that the research was conducted in the absence of any commercial or financial relationships that could be construed as a potential conflict of interest.

## Publisher’s note

All claims expressed in this article are solely those of the authors and do not necessarily represent those of their affiliated organizations, or those of the publisher, the editors and the reviewers. Any product that may be evaluated in this article, or claim that may be made by its manufacturer, is not guaranteed or endorsed by the publisher.
