# Supplementary material for: Creating realistic nerve agent victim profiles for computer simulation of medical CBRN disaster response
Source: Front Public Health. 2023 Jun 29;11:1167706. doi: 10.3389/fpubh.2023.1167706 (PMC10347399; doi:10.3389/fpubh.2023.1167706)
Supplement: Supplementary file 1 [file Table_1.docx]

Supplementary Material

Creating realistic nerve agent victim profiles for computer simulation of medical CBRN disaster response.

De Rouck Ruben^1^, Benhassine Mehdi^2^, Debacker Michel^1^, Dugauquier Christian^3^, Dhondt Erwin^4^, Van Utterbeeck Filip^2^, Hubloue Ives^1^

^1^Research Group on Emergency and Disaster Medicine, Vrije Universiteit Brussel, 103 Laarbeeklaan, Brussels 1090, BELGIUM

^2^Department of Mathematics, Royal Military Academy, 30 Renaissance Avenue, Brussels 1000, BELGIUM

^3^Twenty-third Medical Battalion, Belgian Defense, Tournai, BELGIUM and Belgian delegate in the NATO COMEDS Biological Medical Panel.

^4^DO Consultancy, 759/1Romeinsteenweg, Brussels 1020, BELGIUM

***Correspondence:**Ruben De Rouck
[Ruben.De.Rouck@VUB.be](mailto:Ruben.De.Rouck@VUB.be)

# Supplementary Table 1: Victim profile calculation pseudocode

In the tables below you can find the methodology used to convert the AMedP-8(C) injury profiles to the values used in the appended victim profiles.

Legenda:

GI: Gastro-intestinal severity level (either upper or lower)

N: Neurological severity level

O: Ocular severity level

R: Respiratory severity level

M: Muscular severity level

| Airway | Clear | Not snoring or obstructed |
| --- | --- | --- |
|  | Snoring | M == 3 AND R == 3 |
|  | Obstructed | N == 4 OR R == 4 |

| Respiration pattern | regular | R <= 2 |
| --- | --- | --- |
|  | irregular | R > 2 |

| Respiratory Depth | Normal | R <= 1 |
| --- | --- | --- |
|  | Dyspnea | R > 1 and < 4 |
|  | shallow | R==4 |

| Respiratory rate | 10-20 | R == 0 |
| --- | --- | --- |
|  | 21-29 | R == 1 |
|  | >30 | R == 2 |
|  | 5-15 | R == 3 |
|  | < 5 | R == 4 |

| Oxygen Saturation | 95-100% | R < 0 |
| --- | --- | --- |
|  | 90-100% | R == 1 |
|  | 90-85% | R == 2 |
|  | 80-85% | R == 3 |
|  | <80% | R == 4 |

| Heart Rate | >120 | 1<=N<4 |
| --- | --- | --- |
|  | 60-100 | N==0 |
|  | <60 | N==4 AND R==4 in IP6 |

| Systolic blood pressure | 90-120 | N==0 |
| --- | --- | --- |
|  | >120 | 1<=N<4 |
|  | <90 | N==4 AND R==4 in IP6 |
|  | 0/0 | Dead |

| GCS | V5 | N < 3 |
| --- | --- | --- |
|  | V2-4 | N == 3 |
|  | V1 | N == 4 |
|  |  |  |
|  | M6 | M < 4 |
|  | M2-5 | N == 3 |
|  | M1 | M == 4 or N == 4 |
|  |  |  |
|  | E4 | O < 2 |
|  | E2-4 | 0 >= 2 and N < 4 |
|  | E1 | N == 4 |

| Pupil Size | Normal | O == 0 |
| --- | --- | --- |
|  | Miosis | O > 0 |
